# Comparison of Conventional and Sustainable Lipid Extraction Methods for the Production of Oil and Protein Isolate from Edible Insect Meal

**DOI:** 10.3390/foods8110572

**Published:** 2019-11-13

**Authors:** Myriam Laroche, Véronique Perreault, Alice Marciniak, Alexia Gravel, Julien Chamberland, Alain Doyen

**Affiliations:** Department of Food Sciences, Institute of Nutrition and Functional Foods (INAF), Laval University, Quebec, QC G1V 0A6, Canada; myriam.laroche.6@ulaval.ca (M.L.); veronique.perreault.5@ulaval.ca (V.P.); alice.marciniak.1@ulaval.ca (A.M.); alexia.gravel.1@ulaval.ca (A.G.); julien.chamberland.1@ulaval.ca (J.C.)

**Keywords:** edible insect, *Acheta domesticus*, *Tenebrio molitor*, lipid extraction, protein extraction, protein purification

## Abstract

Edible insects represent an interesting alternative source of protein for human consumption but the main hurdle facing the edible insect sector is low consumer acceptance. However, increased acceptance is anticipated when insects are incorporated as a processed ingredient, such as protein-rich powder, rather than presented whole. To produce edible insect fractions with high protein content, a defatting step is necessary. This study investigated the effects of six defatting methods (conventional solvents, three-phase partitioning, and supercritical CO_2_) on lipid extraction yield, fatty profiles, and protein extraction and purification of house cricket (*Acheta domesticus*) and mealworm (*Tenebrio molitor*) meals. Ethanol increased the lipid extraction yield (22.7%–28.8%), irrespective of the insect meal used or the extraction method applied. Supercritical CO_2_ gave similar lipid extraction yields as conventional methods for *Tenebrio molitor (T. molitor)* (22.1%) but was less efficient for *Acheta domesticus* (*A. domesticus*) (11.9%). The protein extraction yield ranged from 12.4% to 38.9% for *A.* *domesticus*, and from 11.9% to 39.3% for *T. molitor*, whereas purification rates ranged from 58.3% to 78.5% for *A. domesticus* and from 48.7% to 75.4% for *T. molitor*.

## 1. Introduction

The human population is predicted to increase to 9.6 billion by 2050. This population growth combined with an increase in consumer demand for food protein is expected to nearly double the demand for food [1]. With more than 1900 species, edible insects represent an interesting source of protein for human consumption [1]. Edible insects are widely consumed in many parts of the world and some studies have listed the many advantages of entomophagy compared to conventional livestock production systems. The first is the low environmental impact of edible insect production. Compared to conventional livestock meat, the production of edible insects could reduce land use, water consumption, greenhouse gas emissions, and feed conversion [1]. More specifically, and according to a life cycle assessment, Smetana et al. [2] demonstrated that the production of edible insect-based protein powder is two to five times more environmentally beneficial than the production of animal products such as poultry and meat. Moreover, Oonincx et al. [3] demonstrated that the global warming potential and land uses of edible insect production were lower than for other protein sources. The second advantage is related to their high nutritional value. Edible insects are a good source of proteins (55.0%–70.7% w/w for *Acheta domesticus* (*A*. *domesticus*) and 47.2%–65.3% w/w for *Tenebrio molitor* (*T. molitor*), lipids (9.8%–22.8% w/w for *A. domesticus* and 14.9%–43.1% for *T. molitor*), minerals (3.6%–9.1% for *A. domesticus* and 2.7%–4.3% for *T. molitor*) and essential fatty acids, such as linoleic and linolenic acids [4,5,6].

However, in Western countries, many cultural and psychological barriers stand in the way of consumer acceptance of insects as food [1,7]. Nevertheless, to reduce insect food neophobia, previous studies proposed to insert invisible edible insects into various food preparations [8]. This suggests that incorporation of edible insect powder or ingredients into familiar products could increase acceptance and consumption of edible insects [9,10]. Nevertheless, the production of edible insect ingredients, such as protein concentrates or isolates, is a major challenge for food processing optimization. Indeed, because high lipid content can interfere with protein extraction, it is necessary to include a lipid separation step to enhance protein extraction.

Several lipid extraction methods with conventional solvents are described in the literature. Alternative environmentally friendly solvents, such as methanol and ethanol, are also recommended [11]. Three-phase partitioning (TPP) was first reported to be an efficient method for protein extraction, but it is also a good strategy for extracting oil from oleaginous material such as soybeans, seed kernels or almonds, apricots, and rice bran [12]. Finally, supercritical CO_2_ (SC-CO_2_) extraction is another common oil extraction method known for its low operating cost and environmental impact [13]. Several studies have focused on lipid extraction methods applied to insects. Most were performed on wild-caught edible insects using organic solvents for lipid extraction. For example, Tzompa-Sosa et al. [5] extracted lipid fractions from four insect species (*T. molitor*, *Alphitobius diaperinus*, *A. domesticus*, and *Blaptica dubia*) using Soxhlet, aqueous, and Folch extraction methods. Aqueous extraction of lipid gave the lowest lipid yield for all edible insect types, whereas Soxhlet and Folch produced similar yields. Using *T. molitor*, Zhao et al. [13] obtained similar defatting efficiencies (lipid extraction yield of about 30%) using ethanol or a mixture of hexane and isopropanol. The application of supercritical CO_2_ to extractions of *T. molitor* produced a range of defatting from 21% to 95%, depending on duration, temperature, and pressure applied [14]. This method has many advantages, such as being solvent free and reducing oxidation of lipid components [14]. These previous studies demonstrated that the quality of the lipid extract is greatly affected by the extraction procedure. Consequently, the lipid extraction method must be carefully chosen.

In this context, the aims of the present study are to investigate the impact of the six conventional and alternative defatting methods on 1) the lipid extraction yield and lipid profile recovered, and 2) the protein extraction and purification rates.

## 2. Materials and Methods

### 2.1. Insects

House cricket (*A. domesticus*) and mealworm (*T. molitor*) meals were provided by Entomo Farm (Norwood, Ontario, Canada). The global composition of these two edible insects is presented in Table 1. The protein content was determined according to the Dumas method [8,15] (Elementar rapid Micro N cube, Langenselbold, Germany), using a conversion factor of 4.76 to convert nitrogen content to protein content, as determined by Janssen et al. [16] for whole insect meal. The ash content was determined by an incineration method (AOAC 938.08) [17], and the chitin content was determined by the method described by Spinelli et al. [18].

### 2.2. Lipid Extraction Methods

**Figure 1:**
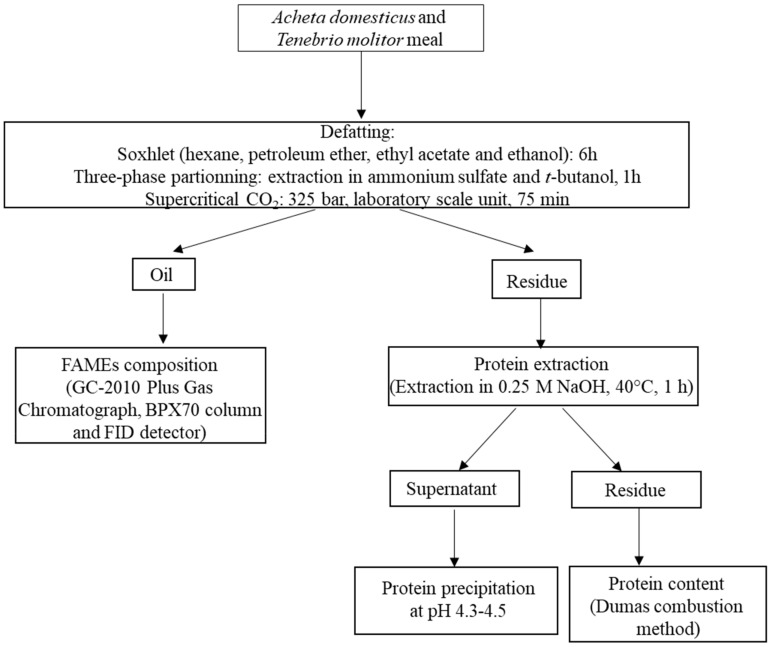
General scheme of experimental procedure.

Figure 1 shows the established protocol described in the following sections. Briefly, *A. domesticus* and *T. molitor* meals were defatted, in triplicate, using one of six different methods. The resulting oils and defatted fractions were recovered and weighed. Between extraction and analysis, these were kept at −20 °C. Oil samples were kept under a nitrogen atmosphere.

#### 2.2.1. Soxhlet Method

Ten grams of insect meal was deposited in a cellulose cartridge (Fisher Scientific catalog number 12-101-100) and the lipids were extracted for 6 h using a Soxhlet apparatus, as described by Tzompa-Sosa et al. [5]. Four different solvents, hexane, petroleum ether, ethyl acetate, and 95% ethanol, were used for the extractions. After each extraction, the solvent was completely removed using a rotary evaporator (R-215, Büchi, Flawil, Switzerland). The lipid extracts and the defatted insect meals were dried at 50 °C in a vacuum oven for a minimum of 5 h and were kept in a desiccator at room temperature before weighing. Lipid extracts were stored at −20 °C.

#### 2.2.2. Three-Phase Partitioning Method

The TPP extraction method applied to insect meals was adapted from Dutta et al. [12] and Panadare and Rathod [11]. Briefly, 10 g of insect flour was mixed with 60 mL of distilled water. The pH of the solution was adjusted to 4.5 with 0.1 M HCl. Next, 20 g of ammonium sulfate and 60 mL of *tert*-butanol (*t*-butanol) were added and the mixture was stirred at 35 °C for 1 h. The mixture was kept at room temperature for 1 h without agitation and centrifuged at 2800× *g* for 5 min. Supernatants were collected in a pre-weighed round-bottom flask and the *t*-butanol was evaporated using a rotary evaporator (R-215, Büchi, Flawil, Switzerland). Extractions were performed in triplicate and oil samples were kept under a nitrogen atmosphere at −20 °C until used.

#### 2.2.3. Supercritical Carbon Dioxide

Supercritical CO_2_ extraction was performed in triplicate at the Centre d’étude des procédés chimiques du Québec (CÉPROCQ) (Montreal, Quebec, Canada) with a laboratory-scale unit, according to the method of Purschke et al. (2017) [14]. Equal masses (15 g each) of insect meal and glass marbles were added to a 500 mL reactor operating at a flow rate of 10 g/mL for 75 min (325 bar, 55 °C). Oil and defatted residue were collected after extraction and weighed. The oil fraction was kept at −20 °C under a nitrogen atmosphere until used.

### 2.3. Characterization of Fatty Acid Methyl Esters (FAMEs) by Gas Chromatography

Oil fractions were derivatized prior to analysis of FAMEs (fatty acid methyl esters). One drop of extracted oil was introduced into a 10 mL screw cap tube. One mL of hexane was added, followed by 0.5 mL of 0.05 M sodium methanolate. The cap was tightened, and the solution was vortexed before heating in a water bath at 40 °C for 15 min. After heating, 2 mL of hexane and 3 mL of saturated NaCl solution were added. The solution was vortexed again and left at rest until the two phases were separated. The organic phase containing FAMEs was collected (supernatant) and filtered through a glass pipette equipped with 1 cm of anhydrous sodium sulfate. The FAMEs were collected in a clean 10 mL screw cap tube. The aqueous phase was washed twice with an additional 2 mL of hexane. The supernatant was collected after phase separation, filtered in a glass pipette as above, and added to the previous 10 mL tube. Another 5 mL of hexane was added for a final hexane volume of 10 mL, and a FAME concentration of about 1 mg/mL.

One microliter of the FAME solution was injected onto a GC-2010 Plus Gas Chromatograph (Shimadzu, Kyoto, Japan) equipped with a BPX70 column (60 m × 0.25 mm internal diameter × 0.25 µm film thickness; SGE, Melbourne, Australia) and a flame ionization detector (FID). Dihydrogen (H_2_), at a flow rate of 1.29 mL/min, was the carrier gas. The injector (split ratio 50:1) was maintained at 250 °C with a purge flow of 3 mL/min. The temperature started at 60 °C for 1 min, increased to 190 °C at 5 °C/min, was maintained at 190 °C for 15 min, then finally increased to 240 °C at 5 °C/min where it was maintained for 1 min, for a total program time of 53 min. The FAMEs were identified by comparing the retention time of the peaks with a commercial standard (C4 to C22 GLC-607; Nu-Chek Prep, Elysian, MN, USA). Analyses were performed in triplicate and the data were analyzed with GCsolution software, version 2.32.00 (Shimadzu, Kyoto, Japan).

### 2.4. Protein Extraction

*A. domesticus* and *T. molitor* proteins were extracted according to Zhao et al. [13]. For both insects, 4 g of defatted or regular (control) meal was added to 60 mL of 0.25 M NaOH. The mixture was agitated for 60 min at 40 °C and centrifuged at 3500× *g* for 20 min at 4 °C. The supernatant was removed, and a second extraction was performed on the residue. Supernatants from the first and second alkaline solubilization were pooled and the pH was adjusted to 4.3–4.5 to induce protein precipitation with 2 M HCl before centrifugation (2500× *g*, 15 min, 4 °C). After protein precipitation, the supernatant was removed, and the residue containing precipitated proteins was washed twice with distilled water at pH 4.5 and centrifuged (2500× *g*, 10 min, 4 °C). The residue and the supernatant were freeze-dried. The powders of the two fractions were analyzed using the Dumas combustion method (Elementar rapid Micro N cube, Langenselbold, Germany). A conversion factor of 5.60 was used to convert the nitrogen percentage to protein content of insect protein extract, as determined by Janssen et al. [16]. This conversion factor is different than that used to determine the protein content of the whole insect meal (Table 1), because the non-protein nitrogen (NPN) content is reduced during the protein extraction [16].

### 2.5. Extraction Yields

For all lipid extraction methods, the oil extraction yield was calculated according to Equation (1):

Oil extraction yield (%) = (m_f_/m_i_) × 100 (1)

where m_f_ is the mass of extracted fat (g) and m_i_ is the mass of crude *A. domesticus* and *T. molitor* meal (g). As proposed by Zhao et al. [14] for *T. molitor*, the extracted fat was expressed as a percentage the initial mass of edible insect meal samples, which means that the most efficient defatting method could not have a 100% lipid extraction yield.

The protein extraction yield was calculated according to Equation (2) [19]:

Protein extraction yield (%) = ((m_r_ × %P_r_)/(m_d_ × %P_d_)) × 100 (2)

where m_r_ represents the mass (g) and %P_r_ represents the protein content (% w/w) of the residue after protein precipitation, and m_d_ and %P_d_ represent the mass (g) and the protein content (% w/w) of defatted *A. domesticus* or *T. molitor* meal (respectively). The protein purity was obtained when determining the nitrogen content of each sample (Elementar rapid Micro N cube, Langenselbold, Germany).

### 2.6. Statistical Analysis

Statistical analyses were performed using Statistical Analysis System (SAS) University Edition, SAS^®^ Studio 3.5 software. A randomized complete block design with six treatments (different extraction methods) in multiple blocks (*n* ≥ 3) was used. For each experiment, the experimental unit was *A. domesticus* or *T. molitor* meal, which received one treatment. Each experiment was performed in triplicate and results were reported as mean ± standard deviation (SD). Tukey tests (α = 0.05) were used as multiple comparison tests. Statistical analyses were done independently for *A. domesticus* and *T. molitor*.

## 3. Results and Discussion

### 3.1. Lipid Extraction Yield and Fatty Acid Composition

Table 2 shows the fat extracted (% w/w of the insect meal) from both insect meals obtained from the different defatting methods. The extraction yields were higher for *T. molitor* (22.1%–28.8% w/w) compared to *A. domesticus* (11.9%–22.7% w/w). This difference is explained by the fact that crude *T. molitor* has a higher fat content than *A. domesticus* (36% w/w [20] vs. 18.6%–22.8% w/w [21]). The results showed that the defatting method significantly impacts the lipid extraction yields for *A. domesticus* (*p* < 0.05), unlike for *T. molitor* (*p* > 0.05). Significant differences were observed between Soxhlet extractions of *A. domesticus*, depending on the solvent used (*p* < 0.05). Ethanol produced the highest lipid extraction rate at 22.7% w/w. The lipid extraction rates for TPP and ethyl acetate were similar, and hexane and SC-CO_2_ were the least efficient methods for lipid extraction with values of 14.6% and 11.9%, respectively. Further optimization of the SC-CO_2_ method using co-solvent might improve the extraction yield in a future work, as shown by Rudyk et al. [22] for the extraction of nonfood and nonpolar compounds.

The fatty acid (FA) profiles of lipids extracted from *A. domesticus* and *T. molitor* meals are shown in Table 3; Table 4, respectively. For both insects, the three most abundant FAs were palmitic acid (C16:0), vaccenic acid (C18:1V), and linoleic acid (C18:2). For *A. domesticus*, C18:2, which is an essential fatty acid, represented 29%–36% of the total FA, while C16:0 and C18:1V comprised 26%–31% and 21%–24% of the total FA, respectively. Stearic acid (C18:0) was the fourth most abundant FA of *A. domesticus*, ranging between 10% and 11% of the total FA.

For *T. molitor*, C18:1V was the dominant FA at 37%–40% of the total FA. The second most abundant *T. molitor* FA was C18:2 (33%–37%), followed by C16:0 (18%–19%). Except for these major FAs, the remaining FAs found in both edible insect lipid extracts were detected at very low relative abundance. Some differences were observed, especially the abundance of C14:0, C17:0, C17:1, and C18:1V, which were higher in *T. molitor* oil extract, or the abundance of C16:0, C18:0, and C18:1 (oleate), which were higher in *A. domesticus* oil extract. Finally, arachidic (C20:0), transvaccenate (C18:1T), petroselinate (C18:1P), gamma-linolenic (C20:3), and docosahexaenoic (C22:6) acids were detected in *A. domesticus* oil, but were absent from *T. molitor* oil.

These results agreed with the work of Tzompa-Sosa et al. [5] who reported that C18:1, C18:2, and C16:0 were the three most abundant FA in four insect species, including *T. molitor* and *A. domesticus*. Mariod et al. [21] findings also supported these results as they indicated that the major FA of adult *A. domesticus* were C18:2 (30%–40%), C18:1 (23%–27%), C16:0 (24%–30%), and C18:0 (7%–11%). They also reported the occurrence of smaller amounts of palmitoleic (C16:1), myristic (C14:0), and linolenic acids (C18:3). Moreover, the proportions of FA obtained from *T. molitor* larvae with SC-CO_2_ were close to the results obtained by Purschke et al. [14], who found 18.46% of C16:0, 2.41% of C18:0, 42.14% of C18:1, and 29.00% of C18:2 for the same treatment (325 bar, 55 °C, 75 min).

There were some differences in the FA profiles, depending on the defatting method used. For both edible insects, Soxhlet extraction with ethanol or TPP were the two least efficient methods for extracting C16:0, whereas SC-CO_2_ was the most efficient. For *A. domesticus* only, Soxhlet extraction with hexane or TPP extracted more C18:0. Ethanol was the least efficient solvent, but there was no significant difference between ethanol and petroleum ether, ethyl acetate, or SC-CO_2_. For both insects, SC-CO_2_ extracted the highest proportion of transvaccenic acid, but no significant difference was observed between Soxhlet extractions with hexane or petroleum ether (or ethyl acetate for *T. molitor*, exclusively). Like the C18:0 extractions, ethanol was the least efficient extraction method for transvaccenic acid but there was no significant difference (*p* > 0.05) between this method and TPP (and ethyl acetate for *A. domesticus* only). Moreover, for both *A. domesticus* and *T. molitor*, TPP and Soxhlet extraction with ethanol were the two defatting methods extracting the most C18:2.

With both insects, extraction of the three most abundant FAs showed that ethanol and TPP, two defatting methods using polar solvents (ethanol and *t*-butanol), are more efficient at extracting C18:2, whereas methods using nonpolar solvents are more efficient at extracting C18:1 and C16:0. These results agree with the findings of Murali et al. [23]. These authors fractionated lipids from *Fusarium* spp. into polar and nonpolar classes using ethanol (polar) and petroleum ether (nonpolar). They found a higher quantity of C18:2 in the polar lipid fraction, whereas the nonpolar lipid fraction contained more C18:1 [23]. The results obtained in this study also agree with the fact that polyunsaturated fatty acids (PUFA) such as C18:2 are relatively more polar than monounsaturated fatty acids (MUFA) such as C18:1V and long-chain saturated FAs such as C16:0 [24].

As explained previously, ethanol produced the highest fat extraction yield. However, for FA quantification, its efficiency was highly dependent on FA relative polarity. These results show that lipids other than FA also affect the fat extraction yield. Indeed, phospholipids, the second most important lipid in insects, after triglycerides, are polar components and therefore very easily solubilized in a polar solvent, such as ethanol, methanol [20,25], or even *t*-butanol, used for TPP extraction. Conversely, triacylglycerols or sterol esters have very low polarity and, so, are soluble in nonpolar solvents, such as hexane and ether [25]. It can therefore be hypothesized that all categories of lipids were present in the same proportions for *T. molitor*, leading to the non-significant differences (*p* > 0.05) in fat extraction yields observed for the defatting methods under study. However, *A. domesticus* meal may have contained a higher proportion of polar lipids since methods using polar solvents such as ethanol and *t*-butanol were significantly more efficient at extracting fat from this insect species. These findings suggest that the most suitable defatting method may differ from one insect to the other, depending on its lipid composition.

### 3.2. Protein Extraction and Purity

Protein extraction experiments were performed on defatted *A. domesticus* and *T. molitor* to generate an enriched protein fraction. Protein concentrations of initial *A. domesticus* and *T. molitor* meals were 53% and 45% w/w on a dry basis, respectively. According to Rumpold and Schulter [6], the protein content of *A. domesticus* meal ranged from 55% to 70%, compared to 47% to 60% for *T. molitor*. These results are higher than those observed in the present study since they used a protein conversion factor of 6.25. However, it was recently suggested by Janssen et al. [16] that this conversion factor overestimates protein concentration because it includes natural nitrogen-containing polysaccharides such chitin, or chitosan, or inorganic nitrogen such as uric acid, urea, or ammonia [26]. Consequently, a protein conversion factor of 5.60, used in this study, seems more appropriate for evaluating the protein content of the protein extract of whole meal and defatted protein extracts [16]. Assuming an overestimation of protein concentration in the literature, the protein concentrations obtained in this study were comparable [13].

The protein extraction yields are presented in Table 5. Extractions performed on defatted *A. domesticus* and *T. molitor* meals were compared to those on the whole meals (Table 1) without defatting to evaluate the importance of defatting to increase protein extraction and protein purity.

Protein extraction was not performed with defatted meals obtained using TPP since ammonium sulfate was used to precipitate the proteins. Ammonium sulfate contains nitrogen, which interferes with the nitrogen analysis to determine protein content. For *A. domesticus*, there was no significant difference (*p* > 0.05) in protein purity between the extraction made with the most polar solvent, ethanol (78.5% ± 2.0%), and the least, hexane (74.7% ± 0.3%). No significant were observed either for *T. molitor*. Yi et al. [7] used aqueous extraction for insect defatting, and after protein extraction of defatted meal following centrifugation, the purity was 50%–61% in the supernatant fraction. The results of this study demonstrated the importance of defatting insect meals and of precipitating proteins after extraction, to obtain protein concentrates with high purity.

Finally, insect protein concentrates and isolates were compared with plant-based proteins. The results are presented in Table 6. The protein extraction yield of insects was quite inferior to vegetal proteins, depending on the extraction method. However, the protein purity of insect extracts can be compared to other protein matrices, but not the whole meal. In fact, the major compositional difference between insect and plant-based matrices is the presence of chitin that has an important structural role in insects [27]. However, chitin in insects, as in crustaceans, is found in a complex matrix made of proteins and fat, rather than in its pure form [27,28]. Consequently, despite the fact that defatting increases the purity of protein extracts, it might be interesting to find a complementary method that would collapse the chitin matrix and, thus, improve protein extraction yields, and the competitiveness of the edible insect industry.

## 4. Conclusions

The impact of six different defatting methods on lipid extraction, fatty acid composition, and protein extraction from two insect meals were investigated. Ethanol provided the highest fat extraction rate. However, according to fatty acid profiles obtained by gas chromatography, ethanol was less effective at extracting relatively nonpolar fatty acids. Consequently, the choice of defatting methodology should be made according to the fat composition of the edible insect used. The lipid extraction method impacts protein extraction, as defatting performed with ethanol produced a higher protein purity for *A. domesticus*. Even if eco-friendly alternatives are available (i.e., SC-CO_2_), there must be a compromise between extraction rate of edible insect macromolecules and sustainability of processing steps. The results also showed that defatting and protein precipitation were important for obtaining good yields and purification rates comparable to results found in the literature for pulse proteins. Furthermore, following this study, it would also be appropriate to optimize the SC-CO_2_ process, notably with the use of co-solvent, to assess the functional properties of these high-purity protein concentrates and to validate their incorporation into functional foods.

## Figures and Tables

**Table 1:** Physicochemical characterization (% w/w on a dry basis) of *Acheta domesticus* (*A. domesticus*) and *Tenebrio molitor (T. molitor)* meals.

Insect Meal	Protein	Ash	Chitin	Other (Sugar, Lipid, etc.)
	% w/w (dry basis)			
*A. domesticus*	53.5 ± 0.3	5.5 ± 0.1	5.9 ± 0.6	35.1 ± 0.6
*T. molitor*	45.7 ± 0.02	4.3 ± 0.1	7.2 ± 0.5	42.9 ± 0.6

**Table 2:** Lipid extraction yield of *A. domesticus* and *T. molitor* meals, according to the defatting method.

Insect Meal	Defatting Method	Extracted Fat (% w/w of Sample Mass)
*A. domesticus*	Soxhlet (Hexane)	14.6 ± 0.1 ^c^
	Soxhlet (Petroleum ether)	14.7 ± 0.2 ^c^
	Soxhlet (Ethyl acetate)	15.1 ± 0.3 ^b^
	Soxhlet (Ethanol)	22.7 ± 2.9 ^a^
	TPP	19.3 ± 2.0 ^ab^
	SC-CO_2_	11.9 ± 1.4 ^c^
*T. molitor*	Soxhlet (Hexane)	25.5 ± 0.1 ^a^
	Soxhlet (Petroleum ether)	24.3 ± 1.2 ^a^
	Soxhlet (Ethyl acetate)	25.7 ± 0.3 ^a^
	Soxhlet (Ethanol)	28.8 ± 5.9 ^a^
	TPP	23.7 ± 2.4 ^a^
	SC-CO_2_	22.1 ± 0.6 ^a^

SC-CO_2_: supercritical CO_2_, TPP: three-phase partitioning. Mean values ± SD with different letters are significantly different (Tukey test, α = 0.05, *n* = 3).

**Table 3:** Relative abundance of fatty acid composition from *A. domesticus* oil.

Fatty Acid		Relative Abundance (u.a.)					
		SH	SP	SA	SE	TPP	SC-CO_2_
Saturated	C14:0	0.81 ± 0.03 ^b^	0.83 ± 0.04 ^b^	0.81 ± 0.05 ^b^	0.69 ± 0.03 ^c^	0.60 ± 0.10 ^c^	0.95 ± 0.03 ^a^
	C16:0	27.70 ± 1.00 ^a,b^	27.50 ± 0.30 ^a,b^	26.90 ± 1.10 ^a,b^	24.60 ± 0.70 ^b^	25.10 ± 2.40 ^b^	29.60 ± 0.30 ^a^
	C17:0	0.29 ± 0.01 ^a^	0.25 ± 0.02 ^a^	0.24 ± 0.03 ^a,b^	0.21 ± 0.01 ^a,b^	0.10 ± 0.10 ^b^	0.22 ± 0.00 ^a,b^
	C18:0	10.30 ± 0.20 ^a^	10.14 ± 0.03 ^a,b^	10.00 ± 0.30 ^a,b^	9.60 ± 0.40 ^b^	10.50 ± 0.10 ^a^	9.92 ± 0.08 ^a,b^
	C20:0	0.60 ± 0.01 ^a^	0.57 ± 0.03 ^a,b^	0.57 ± 0.02 ^a,b^	0.46 ± 0.04 ^c^	0.43 ± 0.06 ^c^	0.50 ± 0.02 ^b,c^
MUFA	C16:1	1.17 ± 0.01 ^a^	1.14 ± 0.03 ^a^	1.12 ± 0.00 ^a,b^	1.10 ± 0.03 ^a,b^	1.02 ± 0.08 ^b^	1.22 ± 0.05 ^a^
	C17:1	-	-	-	-	-	-
	C18:1T	0.15 ± 0.01 ^a^	0.15 ± 0.01 ^a^	0.14 ± 0.01 ^a^	0.12 ± 0.00 ^b^	0.12 ± 0.01 ^b^	0.11 ± 0.00 ^b^
	C18:1P	0.20 ± 0.01 ^b,c^	0.19 ± 0.03 ^b,c^	0.18 ± 0.02 ^c^	0.16 ± 0.02 ^c^	0.40 ± 0.10 ^a,b^	0.52 ± 0.08 ^a^
	C18:1V	21.40 ± 0.70 ^a,b^	21.10 ± 0.50 ^a,b^	20.40 ± 0.40 ^b^	19.50 ± 0.40 ^b^	20.30 ± 1.30 ^b^	22.50 ± 0.40 ^a^
	C18:1	0.58 ± 0.02 ^a^	0.54 ± 0.04 ^a,b^	0.54 ± 0.04 ^a,b^	0.50 ± 0.01 ^a,b^	0.47 ± 0.06 ^b^	0.60 ± 0.02 ^a^
	C20:1	-	-	-	-	0.10 ± 0.00 ^b^	0.11 ± 0.00 ^a^
PUFA	C18:2	30.10 ± 1.10 ^b,c^	29.50 ± 0.50 ^c^	29.30 ± 0.30 ^c^	33.00 ± 0.70 ^a,b^	33.60 ± 2.20 ^a^	27.40 ± 0.50 ^c^
	C18:3	1.52 ± 0.01 ^a^	1.48 ± 0.05 ^a,b^	1.46 ± 0.03 ^a,b^	1.47 ± 0.05 ^a,b^	1.39 ± 0.08 ^b^	1.48 ± 0.03 ^a.b^
	C20:3	0.29 ± 0.01 ^b,c^	0.28 ± 0.02 ^c^	0.30 ± 0.00 ^b,c^	0.43 ± 0.04 ^a^	0.35 ± 0.04 ^b^	0.15 ± 0.01 ^d^
	C22:6	0.14 ± 0.01 ^b^	0.14 ± 0.01 ^b^	0.15 ± 0.02 ^b^	0.15 ± 0.01 ^b^	0.17 ± 0.01 ^a,b^	0.19 ± 0.01 ^a^

C18:1P: methyl petroselinate, C18:1T: methyl transvaccenate, C18:1V: methyl vaccinate, MUFA: monounsaturated fatty acid, PUFA: polyunsaturated fatty acid, SA: Soxhlet (ethyl acetate), SC-CO_2_: supercritical CO_2_, SE: Soxhlet (ethanol), SH: Soxhlet (hexane), SP: Soxhlet (petroleum ether), TPP: three-phase partitioning. Mean values in the same line with different letters are significantly different (Tukey test, α = 0.05, *n* = 3).

**Table 4:** Relative abundance of fatty acid composition from *T. molitor* oil.

Fatty Acid		Relative Abundance (u.a.)					
		SH	SP	SA	SE	TPP	SC-CO_2_
Saturated	C14:0	1.65 ± 0.05 ^b^	1.64 ± 0.02 ^b^	1.60 ± 0.10 ^b^	1.67 ± 0.00 ^b^	1.58 ± 0.01 ^b^	1.72 ± 0.04 ^a^
	C16:0	18.74 ± 0.00 ^a,b^	18.77 ± 0.03 ^a,b^	18.67 ± 0.05 ^a,b^	17.78 ± 0.05 ^c^	18.20 ± 0.40 ^b,c^	19.10 ± 0.20 ^a^
	C17:0	0.42 ± 0.02 ^a^	0.40 ± 0.02 ^a^	0.41 ± 0.00 ^a^	0.41 ± 0.01 ^a^	0.44 ± 0.01 ^a^	0.41 ± 0.00 ^a^
	C18:0	2.30 ± 0.01^c^	2.30 ± 0.01 ^c^	2.33 ± 0.01 ^b,c^	2.80 ± 0.08 ^a^	2.62 ± 0.08 ^a,b^	2.20 ± 0.20 ^c^
MUFA	C16:1	0.93 ± 0.00 ^a^	0.94 ± 0.02 ^a^	0.96 ± 0.00 ^a^	0.95 ± 0.05 ^a^	0.93 ± 0.03 ^a^	0.97 ± 0.02 ^a^
	C17:1	0.13 ± 0.00 ^a^	0.13 ± 0.01 ^a^	-	-	0.11 ± 0.00 ^a^	0.12 ± 0.00 ^a^
	C18:1V	39.74 ± 0.00 ^a^	39.74 ± 0.07 ^a^	39.70 ± 0.03 ^a^	36.94 ± 0.02 ^b^	38.10 ± 0.60 ^b^	39.80 ± 0.30 ^a^
	C18:1	0.31 ± 0.01 ^a^	0.29 ± 0.00 ^a^	0.31 ± 0.01 ^a^	0.30 ± 0.02 ^a^	0.30 ± 0.01 ^a^	0.29 ± 0.01 ^a^
PUFA	C18:2	33.76 ± 0.06 ^c^	33.70 ± 0.05 ^c^	33.80 ± 0.09 ^c^	37.00 ± 0.10 ^a^	34.70 ± 0.50 ^b^	33.40 ± 0.10 ^c^
	C18:3	1.25 ± 0.01 ^a^	1.24 ± 0.01 ^a^	1.28 ± 0.01 ^a^	1.30 ± 0.08 ^a^	1.21 ± 0.02 ^a^	1.27 ± 0.01 ^a^

C18:1P: methyl petroselinate, C18:1V: methyl vaccinate, MUFA: monounsaturated fatty acid, PUFA: polyunsaturated fatty acid, SA: Soxhlet (ethyl acetate), SC-CO_2_: supercritical CO_2_, SE: Soxhlet (ethanol), SH: Soxhlet (hexane), SP: Soxhlet (petroleum ether), TPP: three-phase partitioning. Mean values in the same line with different letters are significantly different (Tukey test, α = 0.05, *n* = 3).

**Table 5:** Mass and protein yield of the residue after protein precipitation of *A. domesticus* and *T. molitor*, according to the defatting method.

Insect Meal	Defatting Method	Protein Extraction Yield (%)	Protein Purity (%)
*A. domesticus*	Whole meal (without defatting)	38.9 ± 1.7 ^a^	58.3 ± 0.5 ^d^
	Soxhlet (Hexane)	32.4 ± 3.8 ^ab^	74.7 ± 0.3 ^ab^
	Soxhlet (Petroleum ether)	33.1 ± 1.0 ^ab^	74.3 ± 2.0 ^b^
	Soxhlet (Ethyl acetate)	31.6 ± 3.0 ^b^	74.4 ± 1.2 ^b^
	Soxhlet (Ethanol)	31.0 ± 4.0 ^b^	78.5 ± 2.0 ^a^
	TPP	ND	ND
	SC-CO_2_	33.7 ± 1.1 ^ab^	70.1 ± 1.9 ^c^
*T. molitor*	Whole meal (without defatting)	39.3 ± 0.8 ^a^	48.7 ± 0.1 ^b^
	Soxhlet (Hexane)	33.7 ± 1.6 ^b^	74.0 ± 2.2 ^a^
	Soxhlet (Petroleum ether)	33.5 ± 1.2 ^b^	72.7 ± 1.5 ^a^
	Soxhlet (Ethyl acetate)	33.2 ± 0.6 ^b^	75.4 ± 0.5 ^a^
	Soxhlet (Ethanol)	33.9 ± 3.7 ^b^	75.3 ± 0.8 ^a^
	TPP	ND	ND
	SC-CO_2_	36.4 ± 1.5 ^ab^	72.7 ± 4.1 ^a^

ND: not determined, SC-CO_2_: supercritical CO_2_, TPP: three-phase partitioning. Mean values ± SD with different letters are significantly different (Tukey test, α = 0.05, *n* = 3).

**Table 6:** Comparison of protein extraction yield and purity between edible insect and several pulse matrices.

Protein Source	Extraction Method	Protein Extraction Yield (%)	Protein Purity (%)	Reference
*A. domesticus* ^1^	Alkaline solubilization and isoelectric precipitation	31.0–38.9	58.3–78.5	
*T. molitor* ^1^	Alkaline solubilization and isoelectric precipitation	33.2–39.3	48.7–75.4	
Pea	Alkali extraction-isoelectric precipitation	62.6–76.7	83.3–86.9	[29]
	Salt extraction	68.2–74.8	71.5–79.3	
	Micellar precipitation	30.7–31.1	81.9–87.8	
Pea	Isoelectric precipitation	55.0	81.7	[30]
	Ultrafiltration	57.1	83.9	
Lentil	Isoelectric precipitation	50.3–62.8	78.2–79.1	
	Ultrafiltration	51.9–60.5	82.7–88.6	
Chickpea	Isoelectric precipitation	53.7–69.1	63.9–73.6	
	Ultrafiltration	50.3–54.7	68.5–76.5	
Flaxseed	Hydrolysis with cellulase followed by isoelectric precipitation	ND	82	[31]

^1^ Data obtained in the present study. ND: not determined.
